# Rif1 interacts with non-canonical polycomb repressive complex PRC1.6 to regulate mouse embryonic stem cells fate potential

**DOI:** 10.1186/s13619-022-00124-9

**Published:** 2022-08-02

**Authors:** Lu Li, Pishun Li, Jiale Chen, Li Li, Yunfan Shen, Yangzixuan Zhu, Jiayi Liu, Lu Lv, Song Mao, Fang Chen, Guang Hu, Kai Yuan

**Affiliations:** 1 Hunan Key Laboratory of Molecular Precision Medicine, Department of Oncology, Xiangya Hospital, Central South University, Changsha, Hunan China; 2 Hunan Key Laboratory of Medical Genetics, School of Life Sciences, Central South University, Changsha, Hunan China; 3 Changjun High School, Changsha, Hunan China; 4 Zhounan High School, Changsha, Hunan China; 5 Epigenetics and Stem Cell Biology Laboratory, National Institute of Environmental Health Sciences, Research Triangle Park, NC 2779 USA; 6 National Clinical Research Center for Geriatric Disorders, Xiangya Hospital, Central South University, Changsha, Hunan China; 7 The Biobank of Xiangya Hospital, Central South University, Changsha, Hunan China

**Keywords:** 2C-like, Totipotency, MERVL, Rif1, PRC1.6

## Abstract

Mouse embryonic stem cells (mESCs) cycle in and out of a transient 2-cell (2C)-like totipotent state, driven by a complex genetic circuit involves both the coding and repetitive sections of the genome. While a vast array of regulators, including the multi-functional protein Rif1, has been reported to influence the switch of fate potential, how they act in concert to achieve this cellular plasticity remains elusive. Here, by modularizing the known totipotency regulatory factors, we identify an unprecedented functional connection between Rif1 and the non-canonical polycomb repressive complex PRC1.6. Downregulation of the expression of either Rif1 or PRC1.6 subunits imposes similar impacts on the transcriptome of mESCs. The *LacO-*LacI induced ectopic colocalization assay detects a specific interaction between Rif1 and Pcgf6, bolstering the intactness of the PRC1.6 complex. Chromatin immunoprecipitation followed by sequencing (ChIP-seq) analysis further reveals that Rif1 is required for the accurate targeting of Pcgf6 to a group of genomic loci encompassing many genes involved in the regulation of the 2C-like state. Depletion of Rif1 or Pcgf6 not only activates 2C genes such as *Zscan4* and *Zfp352*, but also derepresses a group of the endogenous retroviral element *MERVL*, a key marker for totipotency. Collectively, our findings discover that Rif1 can serve as a novel auxiliary component in the PRC1.6 complex to restrain the genetic circuit underlying totipotent fate potential, shedding new mechanistic insights into its function in regulating the cellular plasticity of embryonic stem cells.

**Supplementary Information:**

The online version contains supplementary material available at 10.1186/s13619-022-00124-9.

## Background

As metaphorized in Waddington’s epigenetic landscape, the fate potential of mammalian early embryonic cells is increasingly straitened as development proceeds, from totipotency to pluripotency, all the way to terminal differentiation (Eckersley-Maslin et al. 2018; Xu et al. 2021). The molecular networks underlying this narrowing of differentiation potency have been under intensive investigations, leading to the ground-breaking rejuvenation of differentiated cells back to the pluripotent state (Takahashi and Yamanaka 2006). Sitting atop the Waddington’s landscape, cells of totipotency have received increasing attention in recent years because of their ability to produce a complete organism from a single cell (Ishiuchi and Torres-Padilla 2013; Le et al. 2020; Zhou and Dean 2015). One milestone discovery in this field is that the mouse embryonic stem cells (mESCs) in culture are not uniform, with a small population shifting in and out of a 2-cell (2C)-like totipotent state, as reflected by the transient expression of the 2C stage-specific endogenous retroviral element *MERVL* (Macfarlan et al. 2011; Macfarlan et al. 2012). Subsequent studies have further uncovered a set of characteristic features related to this totipotent state. At the epigenetic level, the 2C-like cells display attenuated higher-order chromatin organization, reduced global DNA methylation, and increased chromatin accessibility. Their histones are more mobile and harbor higher levels of active histone modifications, and their chromocenters are less organized relative to the pluripotent mESCs (Genet and Torres-Padilla 2020). These distinct epigenetic features coincide with a unique transcriptional program reminiscent of that of the 2C stage embryo, with downregulated pluripotent factors such as OCT4, SOX2, and NANOG, and upregulated 2C stage-specific genes (2C genes) including *Zfp352*, *Eif1a,* and the *Eif1a*-like cluster, the *Zscan4* cluster, as well as the repetitive elements *major satellites* and the aforementioned endogenous retrovirus *MERVL* (Eckersley-Maslin et al. 2018; Genet and Torres-Padilla 2020; Lu and Zhang 2015; Xu et al. 2021; Zhou and Dean 2015).

To date, many regulators have been identified to promote or repress the totipotent state. The transcription factor DUX, which can directly activate *MERVL* and *Zscan4*, is both necessary and sufficient to induce the 2C-like totipotency (De Iaco et al. 2017; Eckersley-Maslin et al. 2016; Hendrickson et al. 2017; Whiddon et al. 2017; Yang et al. 2020). Other factors upstream of *DUX*, such as Dppa2, Dppa4, and NELFA, can also activate the 2C program (De Iaco et al. 2019; Eckersley-Maslin et al. 2019; Hu et al. 2020; Yan et al. 2019). On the other hand, genome-wide knockdown screens have identified a vast array of repressors whose downregulation promotes the emergence of the 2C-like cells. These repressors include histone chaperone CAF1 complex, acetyltransferase Tip60/Ep400 complex, H3K9 methyltransferase Setdb1 and its binding proteins Trim28 and Atf7ip, DNA methyltransferase Dnmt1, chromosomal protein Smchd1, transcription factor Myc, RNA N(6)-methyladenosine (m^6^A) modification reader Ythdc1, RNA binding protein Lin28, and components in the post-translational modification SUMOylation pathway (Cossec et al. 2018; Fu et al. 2019b; Huang et al. 2021; Ishiuchi et al. 2015; Liu et al. 2021; Rodriguez-Terrones et al. 2018; Sun et al. 2022; Theurillat et al. 2020; Wu et al. 2020; Yan et al. 2019; Yang et al. 2015).

Several members of the polycomb-group proteins have been linked to the regulation of cell fate potential. The polycomb repressive system is conserved in the five major animal lineages, and in mammals, it has diversified into canonical (cPRC1) and non-canonical complexes (ncPRC1) to regulate a plethora of cellular processes (Schuettengruber et al. 2017). The ncPRC1 can be further divided into six subcomplexes PRC1.1–1.6. Each contains a distinct Pcgf subunit (Gao et al. 2012). The PRC1.6 complex, consisting of multiple subunits including Pcgf6, RNF2, RYBP, L3mbtl2, Mga, Max, and E2F6, is known to inhibit meiotic entry of embryonic cells by targeting meiosis and germline genes (Dahlet et al. 2021; Endoh et al. 2017; Liu et al. 2020; Maeda et al. 2013; Mochizuki et al. 2021; Suzuki et al. 2016; Uranishi et al. 2021). Depletion of PRC1.6 subunits such as Pcgf6 or L3mbtl2 results in multiple defects in embryonic development, including failure of gastrulation, abnormal axis development, and embryonic lethality (Endoh et al. 2017; Liu et al. 2020; Qin et al. 2012). Besides, Pcgf6 is reported to exert essential functions in maintaining pluripotency of embryonic stem cells (Zhao et al. 2017). Of note, knockdown many subunits in the PRC1.6 complex, such as Pcgf6, RNF2, RYBP, Mga, Max, and L3mbtl2, can significantly increase the proportion of 2C-like cells in mESCs, indicating that the PRC1.6 complex is also indispensable for the control of totipotent state (Cossec et al. 2018; Li et al. 2017; Rodriguez-Terrones et al. 2018).

First characterized as a Rap1-interacting factor participating in transcriptional silencing and regulation of telomeres (Hardy et al. 1992), Rif1 has multiple functions ranging from control of replication timing (Cornacchia et al. 2012; Foti et al. 2016; Gnan et al. 2021; Klein et al. 2021; Yamazaki et al. 2013; Yamazaki et al. 2012), promotion of non-homologous end joining (NHEJ) during the repair of DNA double-strand breaks (DSBs) (Chapman et al. 2013; Gupta et al. 2018; Mirman et al. 2018; Noordermeer et al. 2018), to decatenation of DNA bridges in mitosis (Bhowmick et al. 2019; Hengeveld et al. 2015; Zaaijer et al. 2016). We have identified Rif1 as a repressor of the expression of *MERVL* in a previous shRNA candidate screen (Li et al. 2017). Rif1 can bind many endogenous retroviruses and silence their transcription by recruiting a panel of epigenetic regulators. Depletion of Rif1 activates *MERVL* and many genes specifically expressed at the 2C embryo stage (Li et al. 2017), suggesting that it acts as a barrier during the transition from pluripotency to totipotency. However, it is still not clear how Rif1 functions in concert with other totipotency factors to regulate the cellular plasticity of embryonic stem cells.

In this report, by comparing the transcriptomic dynamics after downregulation of the known totipotency regulators, we reveal a novel link between Rif1 and the PRC1.6 complex. Rif1 interacts with Pcgf6, stabilizing the PRC1.6 complex and targeting it to a group of genomic loci involved in the regulation of the 2C-like state. Our finding sheds new light on the complex genetic circuit underlying the potency switch that may translate into improved reprogramming of somatic cells for better therapeutic uses.

## Results

### Transcriptomic correlation analysis reveals functional modules regulating mESCs fate potential

**Fig. 1:**
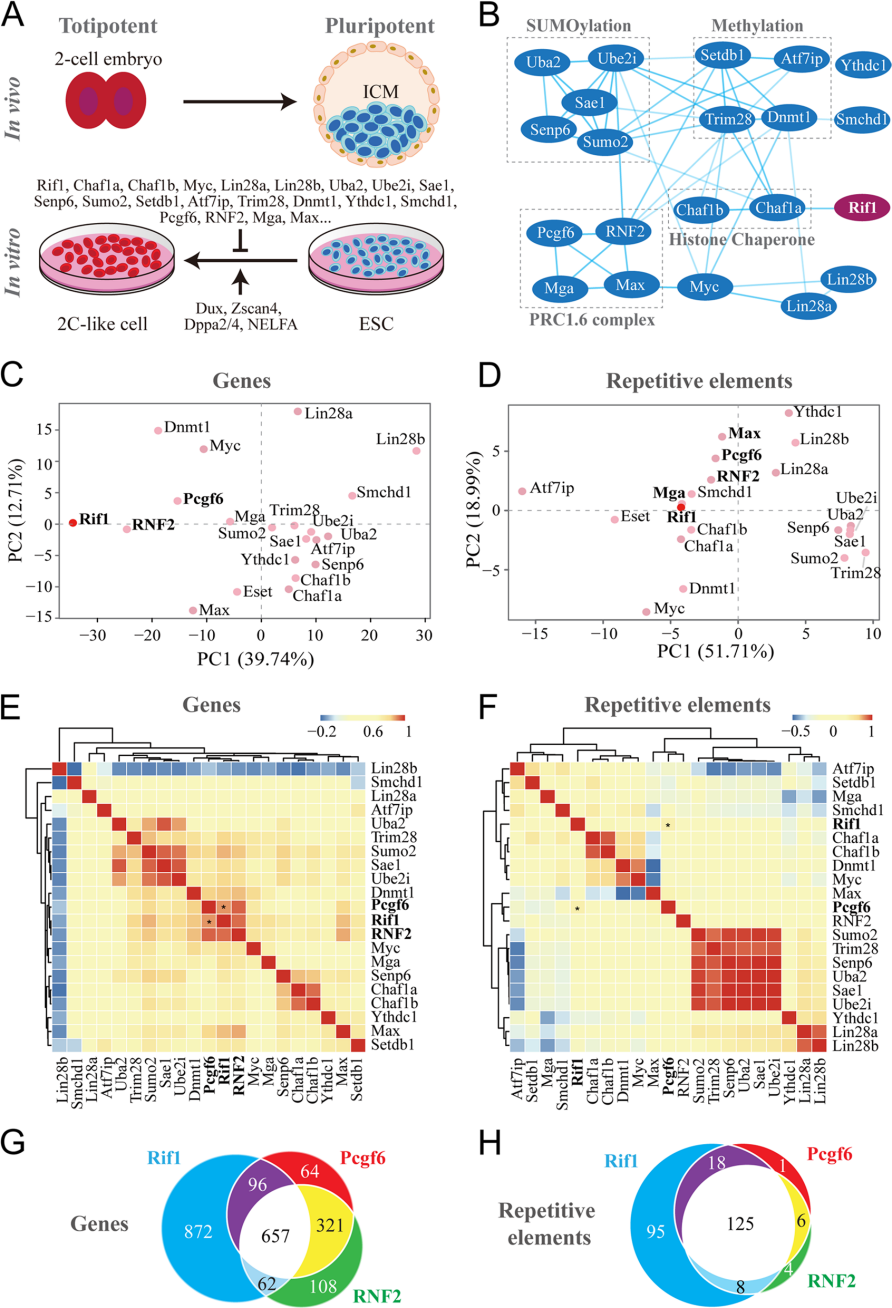
Transcriptomic correlation analysis reveals a functional link of Rif1 to RNF2 and Pcgf6. **A** Known regulators controlling the pluripotency-to-totipotency transition of mESCs. **B** STRING visualization of the protein-protein interaction network of the previously reported 21 repressors of totipotency. **C** Principal component analysis (PCA) of gene expressions of the mESCs with depletion of the indicated genes. **D** PCA based on the expressions of repetitive elements. **E** Correlation matrix showing the unbiased and pairwise comparisons of the global changes in gene expression upon depletion of the indicated genes in mESCs. **F** Correlation matrix generated with the transcriptional changes of repetitive elements upon depletion of the indicated genes in mESCs. Color bars represent Pearson correlation coefficient. **G** Venn diagram illustrating the differentially expressed genes (adjusted *p*-value < 0.05 and |Log_2_ Fold Changes (FC)| > 1) in the mESCs depleted of Rif1, Pcgf6, or RNF2. **H** Venn diagram of the differentially expressed repetitive elements (adjusted *p*-value < 0.05 and |Log_2_ FC| > 1)

Many proteins have been identified to regulate the transitions between totipotency and pluripotency (Fig. 1A). To situate Rif1 in the regulatory circuit governing the metastable fate potential of mESCs, we first assembled the 21 repressors of the 2C-like totipotent state into a functional protein-protein interaction (PPI) network using STRING. Proteins belonging to the same complex (PRC1.6 complex) or involved in similar biological pathways such as SUMOylation and methylation were clustered, forming distinct functional modules. However, Rif1 was rather peripheral in the PPI network, with only a potential connection to the histone chaperone Chaf1a (Fig. 1B). We then collected and compared the transcriptomic data of mESCs after depletion of each of the known repressors. Although downregulating the expression of these repressors could all facilitate the pluripotency-to-totipotency transition, principal component analyses based on the expression of either genes or repetitive elements both revealed significant heterogeneity among these totipotency-like cells (Fig. 1C-D, Supplementary Table 1). Of note, the global transcriptomic changes brought about by the depletion of Rif1 showed a certain degree of similarity to that caused by the knockdown of RNF2, the catalytic subunit of the PRC1 complex harboring the monoubiquitination activity toward lysine 119 of histone H2A (H2AK119ub). We further performed correlation analyses on the transcriptomic data, and we observed strong correlations among the components of the SUMOylation pathway and detected two additional smaller clusters. One contained Chaf1a, Chaf1b, and Senp6, and the other encompassed Rif1, Pcgf6, and RNF2 (Fig. 1E). Since the repetitive portion of the genome is contributing significantly to the genetic wiring of stem cell potency (Fu et al. 2019a; Schlesinger and Goff 2015), we also performed correlation analysis using the expression data of repetitive elements (Fig. 1F). The results were more or less consistent with that generated with coding genes. Proteins involved in the SUMOylation pathway again showed the strongest correlations, and the histone chaperone subunits Chaf1a and Chaf1b were clustered to each other. Although Rif1 in this analysis was not included in any of the clusters, it still manifested a marked correlation with Pcgf6, the characteristic subunit of the PRC1.6 complex.

To directly evaluate the relationship between Rif1 and the PRC1.6 complex, we performed pairwise correlation analyses using the transcriptomic data from mESCs downregulated of Rif1 or core components of the PRC1.6 complex. Knockdown of either RNF2 or Pcgf6 in mESCs resulted in transcriptional changes of both coding genes and repetitive elements highly correlative to that caused by depletion of Rif1 (Fig. S1A-D). We further compared the differentially expressed genes and repetitive elements among these groups (Fig. 1G-H). Of 1138 differentially expressed genes in the mESCs knocked down of Pcgf6, 753 showed consistent changes upon Rif1 depletion. Similarly, 719 out of 1148 differentially expressed genes caused by RNF2 knockdown displayed concomitant changes in the Rif1 depleted mESCs (Fig. 1G). The overlaps of the differentially expressed repetitive elements among these groups were even more dramatic. More than 90% of the Pcgf6- or RNF2-regulated repetitive elements, 143/150 or 133/143, respectively, were also responsive to the depletion of Rif1 (Fig. 1H).

### Rif1 interacts specifically with Pcgf6

**Fig. 2:**
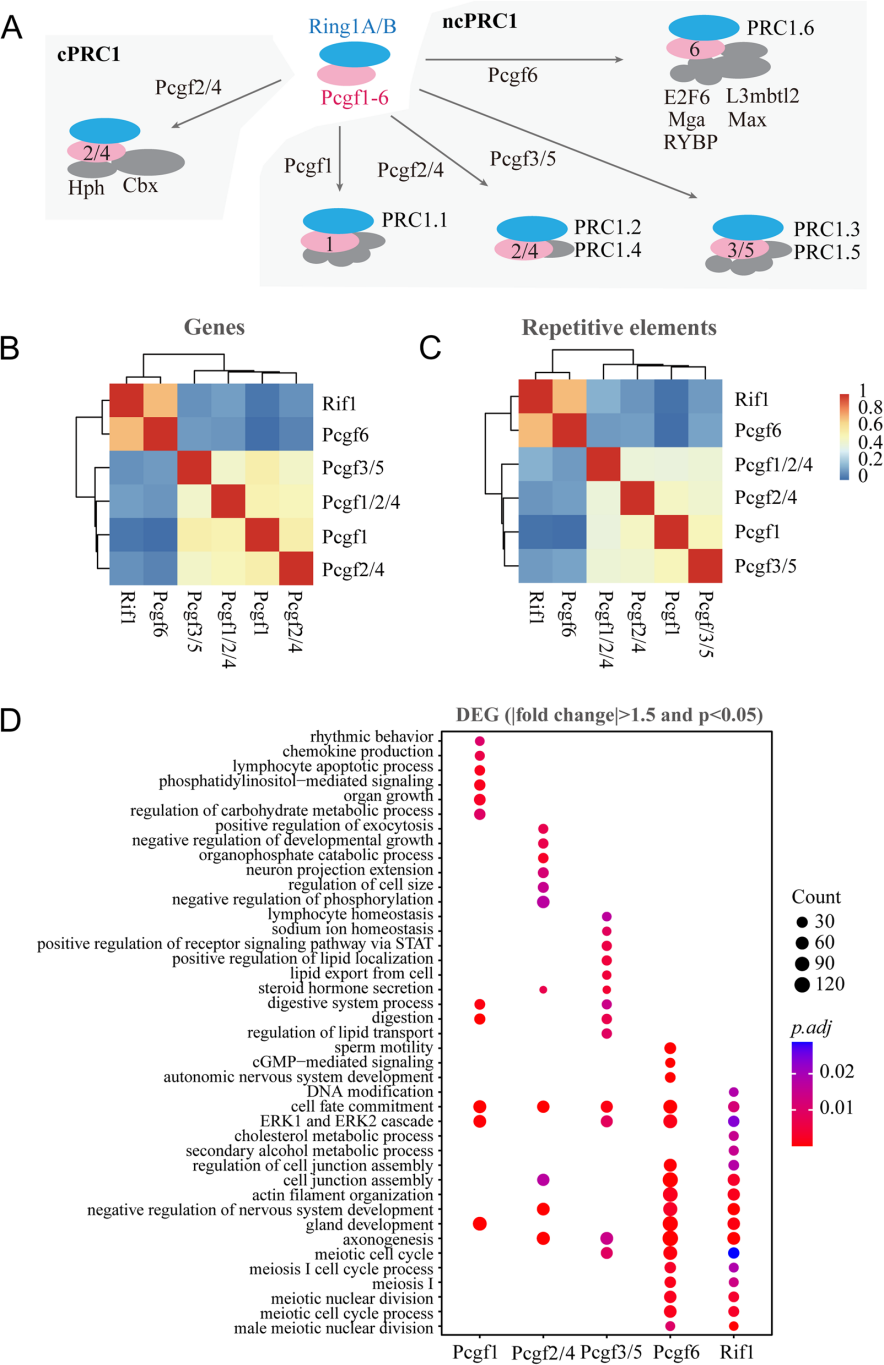
Similar transcriptomic changes caused by depletion of Rif1 or Pcgf6. **A** Schematic illustration of the compositions of the canonical PRC1 (cPRC1) and non-canonical PRC1 (ncPRC1) complexes. **B-C** Correlation analysis of the differentially expressed genes **(B)** or repetitive elements **(C)** in the mESCs depleted of the indicated Pcgf proteins or Rif1. Color bar represents Pearson correlation coefficient. **D** Gene Ontology (GO) analysis of differentially expressed genes (|FC| > 1.5, *p* < 0.05) among the mESCs depleted of Rif1 or the indicated Pcgf proteins. Bubble color indicates the adjusted *p* value, and bubble size represents the number of genes in each category

The analyses of transcriptomic dynamics pointed to a functional link between Rif1 and PRC1.6. The PRC1 complex is heterogeneous, containing several subtypes according to the distinct molecular compositions (Gao et al. 2012; Schuettengruber et al. 2017)(Fig. 2A). To interrogate whether the connection of Rif1 to the PRC1 is specific to the PRC1.6 subcomplex, we collected and analyzed the transcriptomic data from the mESCs with single knockout (KO) of Rif1, Pcgf1, or Pcgf6, double KO of Pcgf2/4 or Pcgf3/5, and triple KO of Pcgf1/2/4 (Scelfo et al. 2019). Both the transcriptional changes of genes and repetitive elements demonstrated a specific correlation between Rif1 and Pcgf6 (Fig. 2B-C). We further performed gene ontology (GO) enrichment analysis on these differentially expressed genes induced by the depletion of Rif1 or the different Pcgf proteins. The results revealed that the depletion of Rif1 or Pcgf6 influenced a similar set of genes enriched in cell fate commitment, cell junction assembly, development of nervous system, as well as multiple meiotic processes, which were markedly different from that influenced by the other Pcgf proteins (Fig. 2D, Supplementary Table 2).

**Fig. 3:**
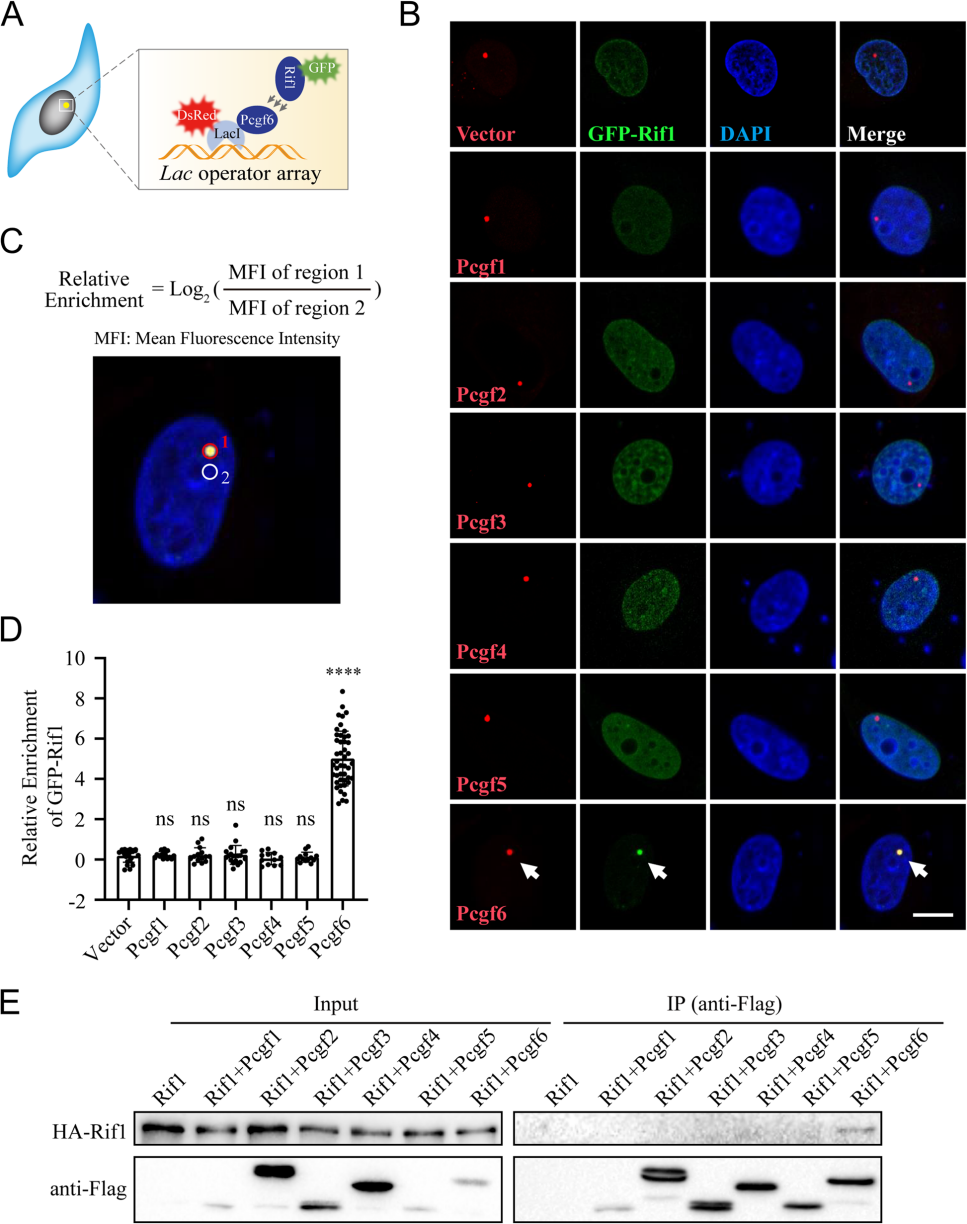
Specific interaction between Rif1 and Pcgf6. **A** Schematic illustrating the *LacO*-LacI system used for detection of protein-protein interactions. **B** The *LacO*-LacI induced ectopic colocalization experiments reveal a specific interaction between Rif1 and Pcgf6. GFP-Rif1 is shown in green, LacI-DsRed fused with the indicated bait proteins are shown in red. DNA is stained with DAPI (blue). Bar: 10 μm. **C** The method to calculate the relative enrichment of GFP-Rif1. **D** The relative enrichment of GFP-Rif1 in the indicated experimental groups. ns: not significant, **** *p* < 0.0001 by t-test, Error bars represent SD, *n* = 12–45 cells per group. **E** Coimmunoprecipitation of HA-Rif1 with different Flag-tagged Pcgf proteins ectopically expressed in HEK293T cells

To explore if there is a physical interaction between Rif1 and Pcgf6, we utilized the *LacO*-LacI induced ectopic colocalization system, in which the bait protein is fused to DsRed-tagged LacI and the prey protein tagged with GFP (Gui et al. 2020). The binding of LacI to the *LacO* array integrated into the genome of U2OS cells concentrates the bait protein, which further recruits the prey protein to manifest an ectopic colocalization of red and green fluorescent signals (Fig. 3A). We used the Pcgf proteins as the baits, and Rif1 tagged with GFP as the prey. When co-transfected into the U2OS cells harboring the *LacO* array, all the bait proteins formed bright nuclear puncta. The puncta of Pcgf6 effectively recruited the prey GFP-Rif1. On the contrary, the puncta formed by other Pcgf proteins failed to do so (Fig. 3B). We quantified the colocalization by calculating the relative enrichment of GFP-Rif1 in the region where the bait protein formed bright puncta (Fig. 3C). The results showed that only Pcgf6 could effectively enrich GFP-Rif1 (Fig. 3D). We further validated this specific interaction by co-immunoprecipitation in HEK293T cells ectopically expressing HA-tagged Rif1 and Flag-tagged Pcgf proteins (Fig. 3E). HA-Rif1 was detected only in the Flag-Pcgf6 immunoprecipitants, indicating a specific interaction between Rif1 and Pcgf6.

**Fig. 4:**
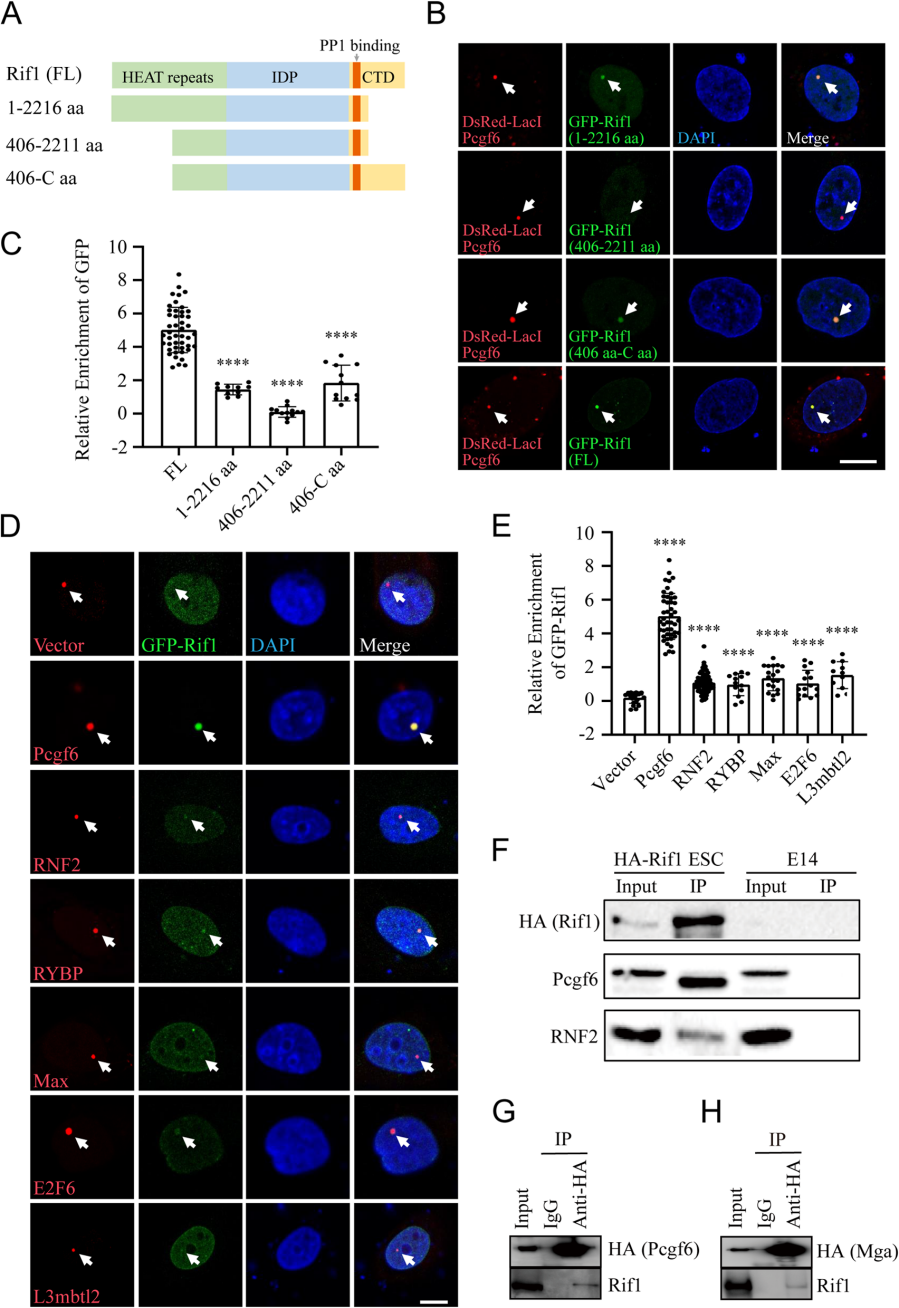
Interactions between Rif1 and other components in the PRC1.6 complex. **A** Schematic of the full-length and truncations of Rif1 proteins. **B** The *LacO*-LacI induced colocalization experiments reveal that both the N- and C-terminal regions of Rif1 can interact with Pcgf6. The full-length and truncations of Rif1 are shown in green, LacI-DsRed fused with Pcgf6 is shown in red, and DNA is stained with DAPI (blue). Bar: 10 μm. **C** The relative enrichment of GFP signals in the indicated experimental groups. **** *p* < 0.0001 by t-test, Error bars represent SD, *n* = 10–45 cells per group. **D** The *LacO*-LacI induced ectopic colocalization experiments reveal that Rif1 can interact with other components in the PRC1.6 complex. GFP-Rif1 is shown in green, LacI-DsRed fused with the indicated bait proteins are shown in red. DNA is stained with DAPI (blue). Bar: 10 μm. **E** The relative enrichment of GFP-Rif1 in the indicated experimental groups. **** *p* < 0.0001 by t-test, Error bars represent SD, *n* = 13–45 cells per group. **F** Coimmunoprecipitation of Pcgf6 and RNF2 with endogenous HA-Rif1. **G-H** Coimmunoprecipitations of Rif1 with endogenously HA-tagged Pcgf6 or Mga in mESCs

To further map the regions on Rif1 that mediate the interaction with Pcgf6, we fused the full-length and different truncated forms of Rif1 to GFP and performed the *LacO*-LacI induced colocalization assay with DsRed-LacI-Pcgf6 (Fig. 4A). While the middle region of Rif1 containing the intrinsically disordered polypeptide (IDP) domain could not be recruited to the Pcgf6 puncta, both the N-terminal and the C-terminal regions of Rif1 were contributing to the interaction with Pcgf6 (Fig. 4B-C).

### Pcgf6 mediates the physical interaction between Rif1 and the PRC1.6 complex

The PRC1.6 complex comprises many subunits, including Pcgf6, RNF2, Max, E2F6, RYBP, and L3mbtl2. To investigate whether Rif1 also interacts with other components of the PRC1.6 complex, we used different subunits of the PRC1.6 complex as the baits and GFP-tagged Rif1 as the prey in the *LacO*-LacI induced colocalization assay (Fig.4D). While the Pcgf6 puncta recruited the most significant amount of GFP-Rif1, all the other PRC1.6 components were able to enrich GFP-Rif1 to varying degrees compared to the control (Fig. 4E), suggesting that Rif1 can interact with the whole PRC1.6 complex. We next examined the interactions between endogenous Rif1 and the components of the PRC1.6 using different knock-in (KI) mESCs. The endogenously HA-tagged Rif1 successfully immunoprecipitated Pcgf6 as well as RNF2 (Fig. 4F). Reciprocally, the endogenously HA-tagged Pcgf6 co-precipitated Rif1 (Fig. 4G). Another component of the PRC1.6 complex Mga also showed a detectable interaction with endogenous Rif1 (Fig. 4H).

**Fig. 5:**
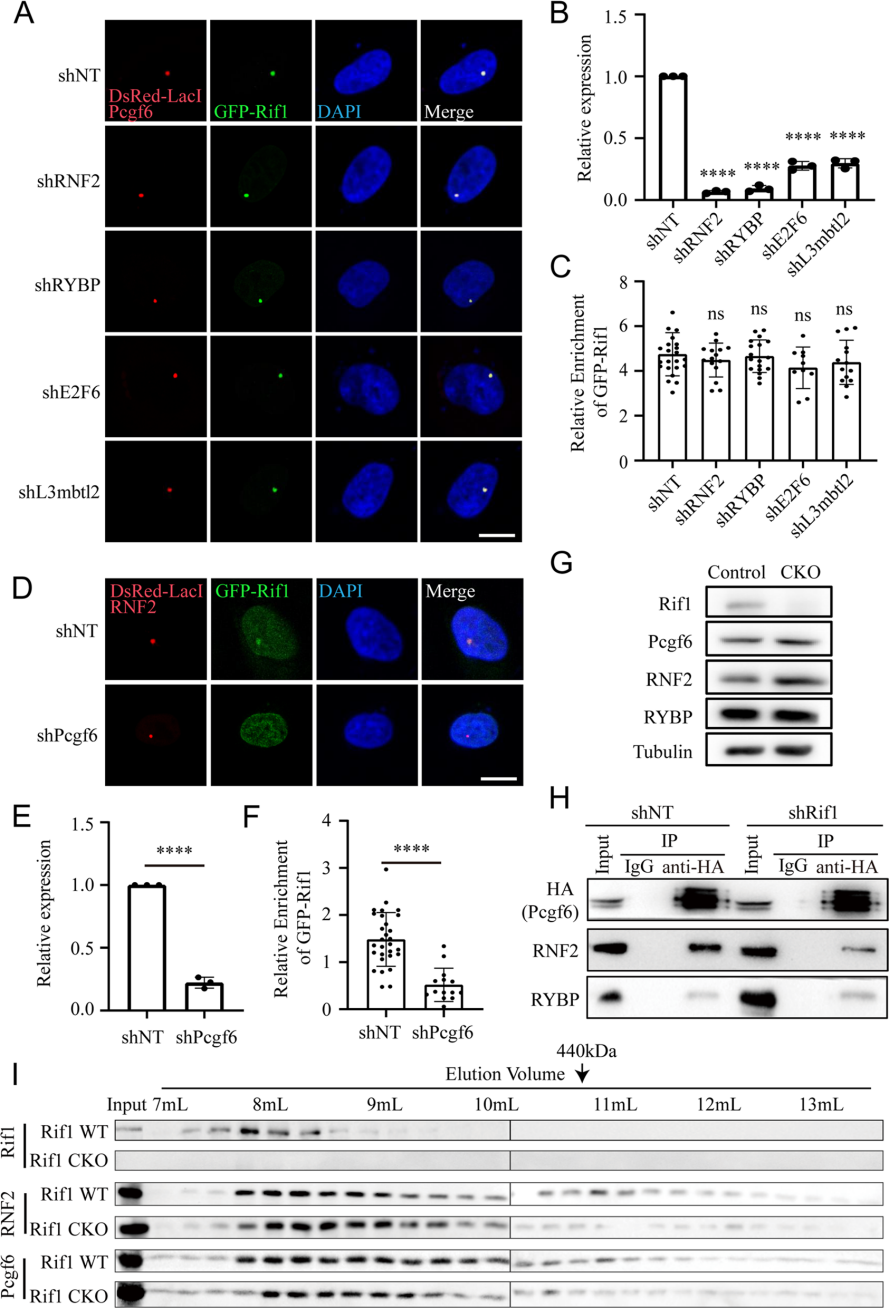
The interaction between Rif1 and PRC1.6 is mediated by Pcgf6. **A** The *LacO*-LacI induced colocalization experiments reveal that knockdown of different components of the PRC1.6 complex has no effect on the interaction between Rif1 and Pcgf6. GFP-Rif1 is shown in green, LacI-DsRed fused with Pcgf6 is shown in red, and DNA is stained with DAPI (blue). Bar: 10 μm. **B** RT-qPCR analysis of the indicated transcripts after the transfection of the corresponding shRNAs in the U2OS-*LacO* cell line. **** *p* < 0.0001 by t-test, Error bars represent SD, *n* = 3. **C** The relative enrichment of GFP-Rif1 in the indicated experimental groups. ns: not significant by t-test, Error bars represent SD, *n* = 10–23 cells per group. **D** The *LacO*-LacI induced colocalization experiments reveal that the interaction between Rif1 and RNF2 is weakened after the knockdown of Pcgf6. GFP-Rif1 is shown in green, LacI-DsRed fused with RNF2 is shown in red, and DNA is stained with DAPI (blue). Bar: 10 μm. **E** RT-qPCR analysis of Pcgf6 after the transfection of shRNAs targeting NT or Pcgf6 in the U2OS-*LacO* cell line. **** *p* < 0.0001 by t-test, Error bars represent SD, *n* = 3. **F** The relative enrichment of GFP-Rif1 in the indicated experimental groups. **** *p* < 0.0001 by t-test, Error bars represent SD, *n* = 29 in shNT group, *n* = 14 in shPcgf6 group. **G** Western blot showing the protein level of the indicated members of the PRC1.6 complex in the presence or absence of Rif1. **H** Coimmunoprecipitation of RNF2 and RYBP with endogenously tagged HA-Pcgf6 in the mESCs treated with shRNA targeting NT or Rif1. **I** Gel filtration of nuclear extracts from the Rif1 WT or Rif1 CKO mESCs. The corresponding elution volumes for each analyzed fraction are labeled. The arrow indicates the approximate elution volume of a 440 kDa protein complex

Given that Pcgf6 manifested the strongest interaction with Rif1, we speculated that the interaction between Rif1 and the PRC1.6 complex was mainly mediated by Pcgf6. To test this, we first used different shRNAs to knockdown the expression of components in the PRC1.6 complex, and evaluated the recruitment of GFP-Rif1 by the Pcgf6 puncta in the *LacO*-LacI induced colocalization assay (Fig. 5A-B). The quantification results of GFP-Rif1 enrichment showed that knockdown of other subunits in the PRC1.6 complex did not attenuate the interaction between Pcgf6 and Rif1 (Fig. 5C). On the contrary, knockdown of Pcgf6 significantly reduced the GFP-Rif1 recruitment by the RNF2 puncta (Fig. 5D-F). Taken together, these results suggested that the interaction between PRC1.6 complex and Rif1 was mediated by Pcgf6.

### Depletion of Rif1 destabilizes the PRC1.6 complex

We next investigated if Rif1 was an integral part of the PRC1.6 complex. We induced conditional knockout (CKO) of endogenous Rif1 by treatment of the mESCs with 4-hydroxytamoxifen (4-OHT) as previously described (Li et al. 2017), and examined the protein levels of several components of the PRC1.6 complex. Rif1 protein became undetectable after 4-OHT treatment, whereas the expression of Pcgf6, RNF2, or BYBP was largely unaltered (Fig. 5G). We next performed immunoprecipitation to investigate the interactions between Pcgf6 and other components in the PRC1.6 complex upon knockdown of Rif1. The endogenously HA-tagged Pcgf6 could co-immunoprecipitate RNF2 and RYBP in the control mESCs. In the Rif1 knockdown mESCs, the interaction between Pcgf6 and RNF2 was compromised (Fig. 5H). We further performed gel filtration experiments in the presence or absence of Rif1 to probe the intactness of the PRC1.6 complex. In the control nuclear extracts, Rif1 was co-eluted with RNF2 and Pcgf6, whereas in the Rif1-depleted nuclear extracts, Rif1 became undetectable and concomitantly, the elution volumes of RNF2 and Pcgf6 were slightly increased, suggesting a decrease in the size of the PRC1.6 complex (Fig. 5I). Taken together, these results suggested that Rif1 was a novel auxiliary component of the PRC1.6 complex.

### Rif1 modulates the genomic distribution of the PRC1.6 complex

**Fig. 6:**
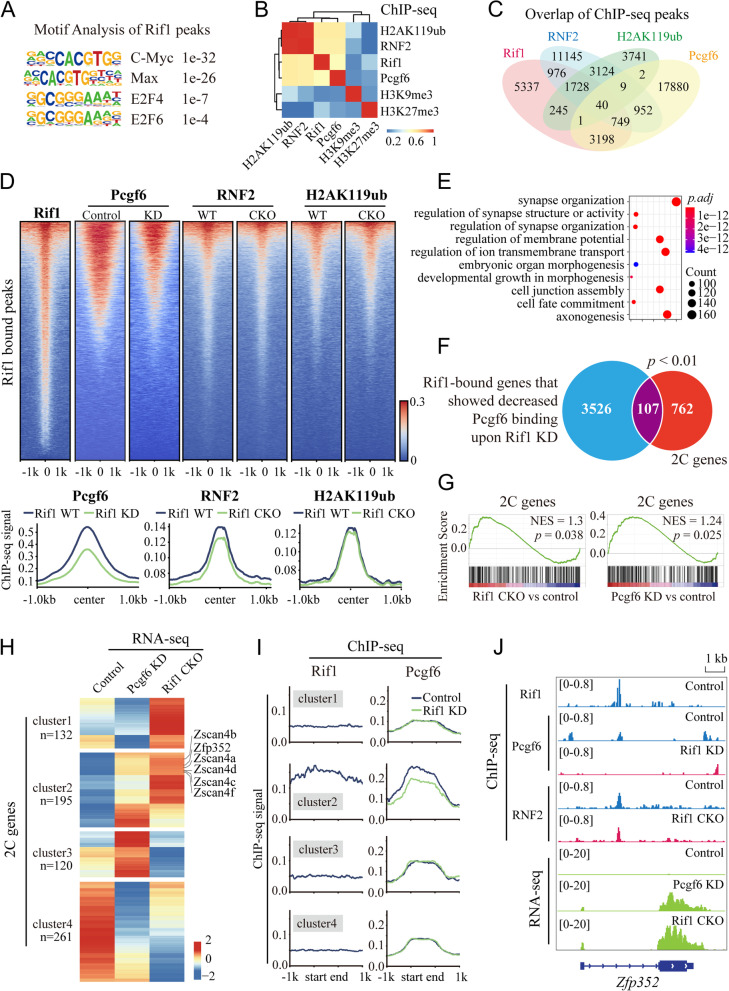
Rif1 modulates the genomic distribution of the PRC1.6 complex and regulates a group of 2C genes. **A** Motif analysis of Rif1-binding sites in mESCs reveals significant enrichment in C-Myc, Max, E2F4, and E2F6 binding motifs. **B** Correlation matrix showing the unbiased and pairwise comparisons of genomic distributions of the indicated proteins or histone modifications in mESCs. Color bar represents Pearson correlation coefficient. **C** Venn diagram comparing the Rif1 binding sites (12,729 peaks) with that of Pcgf6 (22,911 peaks), RNF2 (19,408 peaks), and H2AK119ub (8928 peaks). **D** Top: heatmaps showing the densities of Rif1, Pcgf6, RNF2, and H2AK119ub ChIP-seq reads on the Rif1-bound regions (±1 kb) in control or Rif1 downregulated mESCs. The genomic regions are centered according to the Rif1 ChIP-seq signals in the WT mESCs. Bottom: the averaged ChIP-seq signals of Pcgf6, RNF2, or H2AK119ub on Rif1-bound regions in the presence (blue lines) or absence (green lines) of Rif1. **E** Selected Gene Ontology (GO) analysis results of the genes in the Rif1 and Pcgf6 co-occupied genomic regions which showed decreased Pcgf6 binding upon Rif1 knockdown. **F** Venn diagrams comparing the Rif1-bound genes that showed decreased Pcgf6 binding upon Rif1 knockdown with the 2C genes. Hypergeometric *p* value < 0.01. **G** Gene set enrichment analysis (GSEA) illustrating the upregulation of 2C genes in the mESCs depleted of Rif1 or Pcgf6. The normalized enrichment scores (NES) > 1 and *p* < 0.05 is considered significantly enriched. **H** Heatmap of RNA-seq data showing the expression of the 2C genes in Rif1 CKO and Pcgf6 KD mESCs. The number of 2C genes in each cluster is shown. **I** Averaged Rif1 and Pcgf6 ChIP-seq profiles in the corresponding clusters. **J** Integrative Genomics Viewer (IGV) showing ChIP-seq intensity of indicated proteins and the RNA-seq signals on *Zfp352*

Rif1 is a multi-functional protein that harbors DNA binding activity. To explore its potential function in the PRC1.6 complex, we first analyzed the distribution of Rif1 across the genome using the ChIP-seq data generated previously (Li et al. 2017). Motif analysis revealed that the Rif1-bound peaks also enriched for the consensus binding sequences of the PRC1.6 components, Max and E2F6 (Fig. 6A), suggesting the co-occupancy of Rif1 and PRC1.6 complex. We further performed ChIP-seq experiments for Pcgf6, RNF2, and H2AK119ub, and examined their genomic distributions. The called peaks of Rif1, Pcgf6, RNF2, and H2AK119ub manifested modest correlations (Fig. 6B, Supplementary Table 3). Approximately 1/3 of the Rif1 peaks (*n* = 12,729) were also occupied by Pcgf6 (*n* = 4357), and the overlaps between Rif1 and RNF2 or H2AK119ub were slightly less than that of Pcgf6 (Fig. 6C). To investigate if Rif1 depletion compromises the genomic targeting of the PRC1.6 complex, we analyzed the distributions of Pcgf6, RNF2, and H2AK119ub on the Rif1-bound regions in control and Rif1-downregulated mESCs by ChIP-seq. We observed that the binding of Pcgf6 as well as RNF2 to the Rif1-bound regions were markedly reduced after the downregulation of Rif1 (Fig. 6D, Supplementary Table 3), suggesting that a subset of the genomic distribution of the PRC1.6 complex was dependent on Rif1.

Since Pcgf6 is the characteristic subunit of PRC1.6 and the interaction of the PRC1.6 complex with Rif1 is mainly mediated by Pcgf6, we focused our subsequent analyses on Rif1 and Pcgf6 coregulated genomic regions. We annotated the Rif1 and Pcgf6 co-occupied genomic regions that showed decreased Pcgf6 binding upon Rif1 knockdown and identified 3633 corresponding genes. GO analysis showed that these genes were enriched in biological processes including synapse organization, cell junction assembly, and cell fate commitment (Fig. 6E, Supplementary Table 2). A 2C gene list consisting of 869 genes was generated previously according to their upregulation at the 2C and zygotic genome activation (ZGA) stage in mouse embryonic development (Li et al. 2017). We found that the 2C genes were significantly enriched in the Rif1-bound genes that showed decreased Pcgf6 binding upon Rif1 knockdown (Fig. 6F), suggesting that Rif1 and Pcgf6 functioned in concert to regulate the genetic circuit of totipotency.

### Rif1 and Pcgf6 co-regulate the expression of many 2C genes and MERVL elements

To investigate the cooperation of Rif1 and Pcgf6 in regulating the mESCs fate potential, we performed RNA-seq analysis with mESCs downregulated of Rif1 or Pcgf6. The gene set enrichment analysis (GSEA) revealed that the 2C genes were significantly upregulated in mESCs with reduced expression of either Rif1 or Pcgf6 (Fig. 6G, Supplementary Table 4). We further examined the expression of all the 2C genes using the RNA-seq data, and identified four different clusters (Fig. 6H). Genes in cluster1 showed upregulated expression only in mESCs depleted of Rif1, genes in cluster3 were only activated by the downregulation of Pcgf6, and genes in cluster4 were not upregulated in either condition. We uncovered 195 genes in cluster2 whose expression was consistently upregulated in mESCs downregulated of Rif1 or Pcgf6. Interestingly, genes in cluster2 displayed higher Rif1 and Pcgf6 binding, and the amount of Pcgf6 on these genes was markedly decreased in mESCs depleted of Rif1 (Fig. 6I). We noticed that several 2C stage marker genes, such as *Zfp352* and *Zscan4* were in cluster2. We visualized the ChIP-seq and RNA-seq signals at the *Zfp352* locus, and the results showed that depletion of Rif1 almost eliminated the Pcgf6 peaks, which was accompanied by the significant upregulation of transcription (Fig. 6J).

**Fig. 7:**
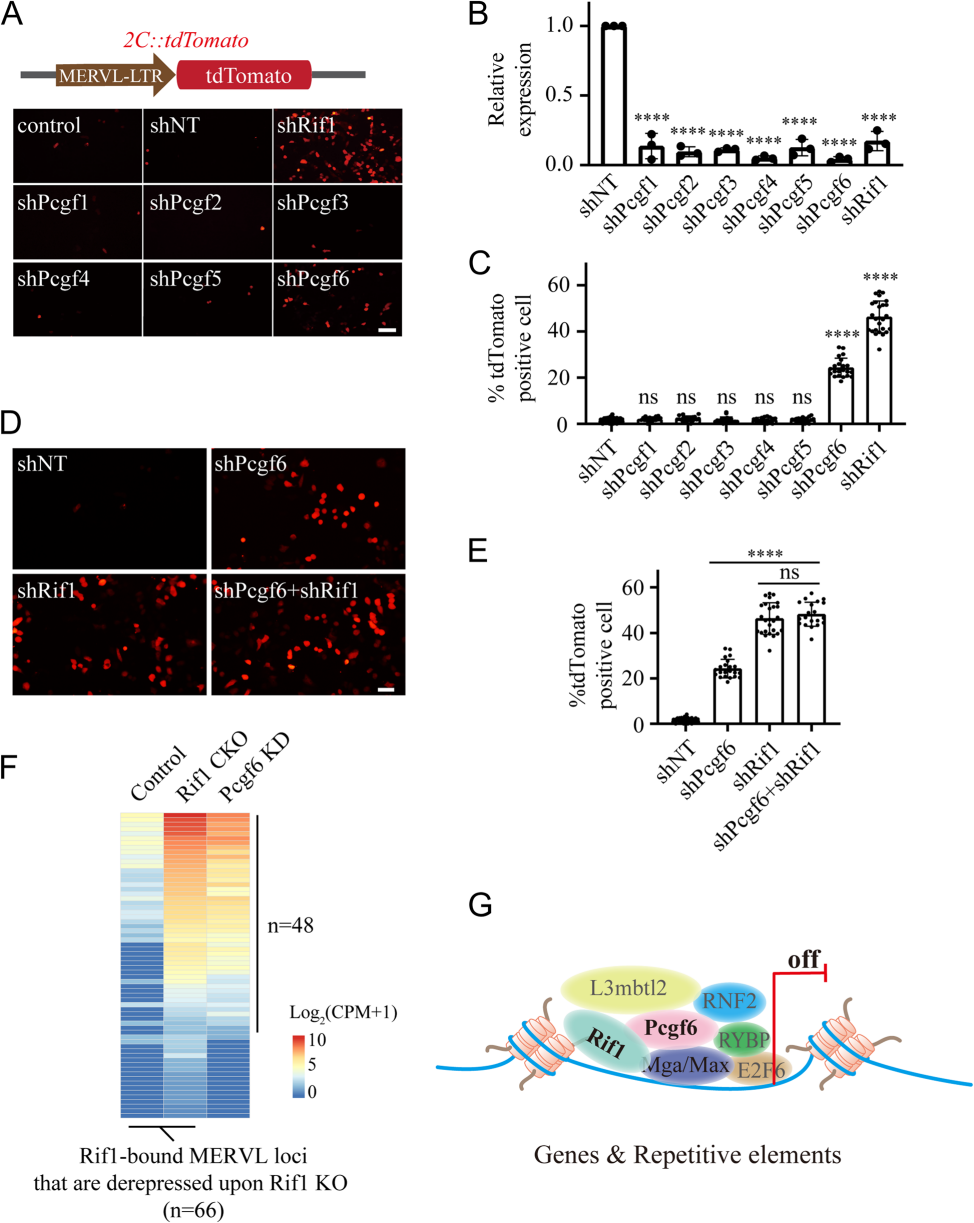
Rif1 and Pcgf6 coregulates a group of MERVL. **A** Top: schematic of the *2C::tdTomato* reporter. Bottom: fluorescent images showing the fraction of tdTomato-positive cells in control or mESCs treated with the indicated shRNA. Control, non-transfected; shNT, non-target shRNA transfected. Bar: 50 μm. **B** RT-qPCR analysis of the indicated transcripts after the transfection of the corresponding shRNAs in the *2C::tdTomato* reporter cell line. **** *p* < 0.0001 by t-test, Error bars represent SD, *n* = 3. **C** The percentages of tdTomato-positive cells in the indicated experimental groups. ns: not significant, **** *p* < 0.0001 by t-test, Error bars represent SD, *n* = 16–26 fields per group. In each field, the number of total cells is approximately 150–350. **D** Fluorescent images showing the fraction of tdTomato-positive cells in control or mESCs treated with the indicated shRNA. shNT, non-target shRNA transfected. Bar: 25 μm. **E** The percentages of tdTomato-positive cells in the indicated experimental groups. ns: not significant, **** *p* < 0.0001 by t-test, Error bars represent SD, *n* = 20–26 fields per group. In each field, the number of total cells is approximately 150–350. **F** Heatmap of RNA-seq data showing the expression levels of the Rif1-bound MERVL loci that are derepressed upon Rif1 depletion (*n* = 66) and their corresponding expression levels in mESCs after Pcgf6 knockdown. A fraction of these MERVL loci (*n* = 48) becomes activated in Pcgf6 KD mESCs. The color bar denotes the Log_2_(CPM + 1). **G** Schematic model of Rif1 as a new auxiliary component of the PRC1.6 complex

The transcriptional activation of repetitive element *MERVL* is a marker for the 2C-like totipotent state, and the *2C::tdTomato* reporter can reflect the transcriptional activity of the long terminal repeat (LTR) controlling the *MERVL* expression. We infected the *2C::tdTomato* mESCs with lentiviruses carrying shRNAs targeting Rif1 or different Pcgf members. Consistent with our previous study (Li et al. 2017), the knockdown of Rif1 substantially expanded the number of tdTomato positive cells (Fig. 7A). Knockdown of Pcgf6 but not the other Pcgf members also significantly increased the tdTomato positive cells (Fig. 7B-C), although the increase was less profound when compared with that induced by Rif1 knockdown. To further examine the cooperation between Rif1 and Pcgf6 in regulating *MERVL*, we simultaneously knocked down Rif1 and Pcgf6 using shRNAs, and compared the effect with that from individual knockdowns (Fig. 7D). While knockdown of Rif1 induced more tdTomato positive cells than that of Pcgf6, the double knockdown showed no additive effect (Fig. 7E), suggesting that Pcgf6 was in the same regulatory pathway as Rif1. Additionally, we analyzed the expression of *MERVL* using our ChIP-seq and RNA-seq data, and unambiguously identified 66 Rif1-bound *MERVL* loci that became transcriptionally activated after Rif1 depletion. 48 of them could also be activated by Pcgf6 knockdown (Fig. 7F, Supplementary Table 4), indicating that these *MERVL* loci were co-regulated by Rif1 and Pcgf6. Taken together, these results suggested that Rif1 and the PRC1.6 complex could form a functional module to control the transition from pluripotency to totipotency by restraining a group of 2C genes and *MERVL* elements.

## Discussion

Comprehensive characterization of the regulatory network driving the dynamic changes of differentiation potentials is of the utmost importance for the decoding of cell fate decisions. With the increasing number of nodes being identified, a complete genetic circuit underpinning the transitions between pluripotency and totipotency is on the horizon (Fu et al. 2020; Fu et al. 2019b; Genet and Torres-Padilla 2020; Le et al. 2020). In this report, we identified a module consisting of Rif1 and PRC1.6 required for the inhibition of the 2C-like totipotent state (Fig. 7G). The Rif1-PRC1.6 module can be involved in the coding of the default developmental trajectory of embryonic cells. When the activators such as DUX are no longer in place, this default program ensures the accuracy and unidirectionality of differentiation. Consistent with this, both Rif1 and Mga in the PRC1.6 complex have been reported to limit mESCs to acquire extraembryonic cell fates (Qin et al. 2021; Zhang et al. 2022). Given their relatively high mRNA abundance in the 2C stage embryo as well as the spontaneously arisen 2C-like cells, the activity of this module is probably harnessed at the protein level. Interestingly, in mice 2C stage embryos, *Rif1*-derived polypeptides are mainly in truncated forms that are likely nonfunctional, and then the full-length Rif1 protein gradually increases as embryos develop to the morula stage (Yoshizawa-Sugata et al. 2021), coinciding with the straitening of cell fate potential. Additionally, protein SUMOylation, which has been implicated in the regulation of 2C-like totipotent state, can modify both Rif1 and PRC1.6 components (Kumar and Cheok 2017; Theurillat et al. 2020). It is plausible that SUMOylation plays a crucial role in controlling the activity of the Rif1-PRC1.6 module.

The non-canonical PRC1 is heterogeneous and encompasses several subcomplexes with distinct molecular compositions (Gao et al. 2012; Schuettengruber et al. 2017). Consistent with previous findings, we were unable to induce 2C-like cells by knockdown of Pcgf1–5 (Fig. 7A-C), suggesting that other ncPRC1 subcomplexes have a limited contribution to totipotency (Rodriguez-Terrones et al. 2018). The PRC1.6 complex comprises Pcgf6, the two core subunits RYBP and RNF2 that are shared among other ncPRC1 subcomplexes, and subunits that are specific to the PRC1.6 including L3mbtl2, Mga, Max, E2F6, and DP1. The genomic targeting of the PRC1.6 complex is cell type- and context-dependent, with combinatorial contributions from Mga-Max, E2F6-DP1, and L3mbtl2 (Huang et al. 2018; Scelfo et al. 2019; Stielow et al. 2018; Uranishi et al. 2021). We uncovered a novel interaction between Rif1 and PRC1.6, which is important for the effective targeting of PRC1.6 to a certain group of genomic loci involved in the control of fate potential in mESCs, further increasing the complexity of its genomic recruiting mechanisms. The functional differences of the PRC1.6 complex resulted from these distinct recruitment mechanisms are of interest to be elaborated in the future. The PRC1.6 complex inhibits premature differentiation of mESCs by repressing germ cell-related genes (Dahlet et al. 2021; Endoh et al. 2017; Liu et al. 2020; Maeda et al. 2013; Mochizuki et al. 2021; Suzuki et al. 2016; Uranishi et al. 2021), and loss of Max results in meiotic entry even in mESCs (Suzuki et al. 2016). Similar to that of Pcgf6, we found downregulation of Rif1 also caused mis-regulation of genes in meiosis-related pathways (Fig. 2D), suggesting that Rif1 can be involved in the control of meiosis onset as well.

Rif1 by itself bears multiple molecular functions, including recognition of G-quadruplex (Kanoh et al. 2015; Masai et al. 2019; Masai et al. 2018), regulation of telomeres (Dan et al. 2014; Hafner et al. 2018; Hardy et al. 1992; Shubin et al. 2021), control of replication timing (Cornacchia et al. 2012; Foti et al. 2016; Gnan et al. 2021; Klein et al. 2021; Yamazaki et al. 2013; Yamazaki et al. 2012), repair of DNA DSBs (Chapman et al. 2013; Gupta et al. 2018; Mirman et al. 2018; Noordermeer et al. 2018), and decatenation of DNA bridges during cytokinesis (Bhowmick et al. 2019; Hengeveld et al. 2015; Zaaijer et al. 2016). Rif1 protein contains two major conserved domains: the N-terminal HEAT-repeats and the C-terminal conserved region. The N-terminal domain is necessary for DNA damage repair, regulation of telomere length, and inhibition of *MERVL* expression (Escribano-Diaz et al. 2013; Li et al. 2017; Shubin et al. 2021; Yoshizawa-Sugata et al. 2021). In addition, the interactions between Rif1 and Setdb1, EZH2, and other methyltransferases also depend on the HEAT-repeats domain (Li et al. 2017). Both the N-terminal and C-terminal regions can bind to G-quadruplex and may be required for stimulation of non-homologous end joining DNA repair (Alavi et al. 2021; Moriyama et al. 2018). The C-terminal region of Rif1 harbors the protein phosphatase 1 (PP1)-binding motifs, which are essential for replication timing and nuclear organization (Alavi et al. 2021; Gnan et al. 2021). Knockdown of Rif1 resulted in a much stronger activation of *MERVL* relative to Pcgf6 (Fig. 7D-F), indicating that it could affect the totipotent state in more than one way. Its interactions with multiple histone methyltransferases such as Setdb1 are likely contributing to this regulation (Li et al. 2017). Interestingly, the PRC1.6 complex collaborates with Setdb1 to achieve precise temporal repression of germline genes in embryonic cells (Mochizuki et al. 2021). Rif1 could be the hub protein orchestrating the dynamic interactions between these different epigenetic machineries to regulate the totipotency genetic circuit. Besides, the 2C embryos, as well as the 2C-like mESCs, undergo rapid telomere lengthening through Zscan4-dependent recombination (Dan et al. 2017), which might involve Rif1’s telomeric function. More intriguingly, the induction of *DUX* and the 2C-like program requires the DNA damage response pathway and the activation of P53 (Atashpaz et al. 2020; Grow et al. 2021), and the 2C marker Zscan4 can bind microsatellite DNA to protect the fragile genomic regions from DNA damage (Srinivasan et al. 2020). It is possible that Rif1 can influence the totipotency via its central function in DNA damage response. Future studies are needed to interrogate this mysterious relationship between DNA damage response and the acquisition of the totipotent fate potential, which could be vital for the development of a better and safer reprogramming protocol of totipotent stem cells for regenerative medicine.

## Conclusions

Our study reported a novel interaction between Rif1 and the non-canonical PRC1.6 complex. Downregulation of Rif1 or members in the PRC1.6 complex resulted in similar transcriptomic changes in mESCs. Rif1 mainly interacted with the Pcgf6 subunit of the PRC1.6 complex, strengthening its integrity. Moreover, Rif1 contributed to the genomic targeting of PRC1.6 to a group of loci, and depletion of either Rif1 or Pcgf6 activated many 2C genes and endogenous retroviral element *MERVL*, suggesting that the Rif1-PRC1.6 module plays an essential role in controlling the cell fate potential of mESCs.

## Methods

### Cell cultures

E14 mouse embryonic stem cells (mESCs) were obtained from the American Type Culture Collection (ATCC). Genetically engineered mESCs, including *Rif1*-CKO, HA-*Rif1* KI, HA-*Pcgf6* KI, HA-*Mga* KI, and 2C::tdTomato reporter cell lines were constructed and cultured in gelatin-coated plates using high-glucose Dulbecco’s modified Eagle’s medium (DMEM, HyClone, SH3002202b) supplemented with 15% ESC-qualified fetal bovine serum (FBS, Vistech, SE200-ES), 0.1 mM 2-mercaptoethanol (Sigma, m3148), 1× non-essential amino acids (NEAA, Gibco, 11140050), and 1000 U/mL of LIF (Millipore, ESG1107). For maintenance, mESCs were cultured in the serum-free ESGRO medium (Millipore, sf001–500p). The U2OS-*LacO* cells (kindly provided by Professor Xuebiao Yao) and HEK293T cells were maintained in high-glucose DMEM supplemented with 10% FBS (Vistech, SE100-B). All cell lines were tested for mycoplasma contamination, and cultured at 37 °C in a humidified incubator containing 5% CO_2_.

### Generation of genetically engineered mESCs

The CRISPR-Cas9 system was used to construct the genetically edited mouse embryonic stem cell lines. The HA-*Rif1* KI cell line was generated in our previous study (Li et al. 2017). For the generation of other HA-tag KI mESCs (HA-*Pcgf6* and HA-*Mga*), the specific guide RNAs (gRNA, Supplementary Table 5) were designed and inserted into the pX330 plasmid. The gRNA plasmid and plasmids containing the homologous recombination (HR) donor sequences (Supplementary Table 5) were co-transfected into E14 mESCs using Lipofectamine 2000 (Invitrogen, 11,668,019). The transfected cells were then replated and individual colonies were picked and screened by PCR. Correctly targeted clones were then amplified and re-screened by Western blot with anti-HA antibody (CST, 3724S). The *Rif1*-CKO cell line was generated as previously described (Li et al. 2017). Briefly, the gRNA plasmid and homologous recombination (HR) donor sequences assembled by inserting two LoxP sites at introns 4 and 7 were co-transfected into Rosa26 CreERT2 mESCs using Lipofectamine 2000 (Invitrogen, 11,668,019). The transfected cells were then replated and individual colonies were picked and screened by PCR. *Rif1*-CKO was initiated with 0.2 μM 4-OHT (Sigma, H6278) for 2 days.

### Plasmids construction and transfection

Primers and oligos used in this study were listed in Supplementary Table 5. For pLKO.1 shRNAs, complementary single-stranded oligos were annealed and cloned as suggested by the RNAi consortium (the BROAD Institute). The *Rif1* full length and truncation fragments were PCR amplified and cloned into pCDNA5-GFP and pHAGE-HA vectors. The cDNA of *Pcgf1–6, RNF2*, *RYBP, Max, E2F6,* or *L3mbtl2* was cloned into the pDsRedC2-LacI and pCDH-CMV-Flag vectors. For lentivirus preparation, HEK293T cells were plated at a density of 2.5 × 10^7^ in 100 mm Petri dishes and cultured overnight. The pLKO.1 shRNA plasmid was co-transfected with the packaging plasmids pPAX2 and pMD.2G using Polyethylenimine Linear (PEI, Polysciences, 24,765). The medium was changed after 8 h, and the viral supernatants were collected 48 h and 72 h post-transfection. For transient expression, cells were transfected using Lipofectamine 2000 (Invitrogen, 11,668,019).

### Immunoprecipitation

Cells were washed with cold DPBS (Biological Industries) for 3 times then harvested and lysed with lysis buffer (50 mM Tris, 150 mM NaCl, 0.5% NP40, 5 mM ethylene diamine tetraacetic acid (EDTA), pH 7.5) supplemented with protease inhibitor cocktail (Sigma, P8340) and phenylmethylsulfonyl fluoride (PMSF, Sigma, P7626) on ice for 1 h. After centrifugation at 17000 g for 40 min at 4 °C, the supernatants were incubated with anti-HA Affinity Matrix (Roche, 11,815,016,001) or anti-Flag M2 Affinity Gels (Sigma, A2220) overnight at 4 °C. The beads were then washed with lysis buffer for 5 times and boiled in sample buffer (50 mM Tris, 2% (w/v) sodium dodecyl sulfate (SDS), 10% (v/v) glycerine, 1% (v/v) 2-mercaptoethanol, 0.01% (w/v) bromophenol blue) at 95 °C for 10 min to elute the protein complex. The concentration of protein was determined using BCA assay (Beyotime, P0009).

### Western blot

Cells were washed with cold DPBS for 3 times, lysed in 2× Laemmli buffer (2% SDS, 20% glycerol, and 125 mM Tris-HCl, pH 6.8) supplemented with protease inhibitor cocktail, and then boiled at 95 °C for 10 min. The protein was separated by SDS-PAGE gel and transferred onto the PVDF membrane (Millipore, IPVH00010). The membrane was then blocked with 5% non-fat milk at room temperature for 1 h and incubated with the indicated primary antibody overnight at 4 °C. After washing with PBST for 3 times, the membrane was incubated with appropriate secondary antibodies (1:10000, Thermo Fisher Scientific) at room temperature for 1 h. The signal was then detected with ECL substrates (Millipore, WBKLS0500). Antibodies used in this study were: anti-HA (CST, 3724S), anti-Flag (CST, 14793S), anti-Rif1 (Abcam, ab13422), anti-Pcgf6 (Lifespan, LS-C158553–400), anti-RNF2 (Active Motif, 39,663), anti-RYBP (Santa Cruz, sc-374,235), and anti-H2AK119ub (CST, 8240S).

### LacO-LacI induced colocalization assay

The pCDNA5-GFP plasmid was co-transfected with pDsRed-LacI plasmid into *LacO*-U2OS cells (with Lac operator array inserted) using Lipofectamine 2000 (Invitrogen, 11,668,019). The medium was changed after 8 h, and the cells were harvested 36 h after transfection. The cells were washed with DPBS for 3 times, fixed in 4% PFA at room temperature for 10 min, and then rinsed in DPBS for 3 times. The cells were stained with DAPI (Sigma, D9542) diluted in PBST (DPBS with 0.05% Triton X-100) for 5 min and washed 3 times with PBST before mounting in SlowFade Diamond mountant (Thermo, S36963). Images were then collected on the Zeiss LSM880 confocal system. The images were analyzed and measured with ZEN 2 blue v2.3 (Zeiss). All experiments were performed three or more times, and representative results were shown.

### Preparation of nuclear extracts and gel filtration

Control mESCs treated with DMSO or *Rif1*-CKO mESCs treated with 4-OHT (0.2 μM) for 2 days were cultured for another 2 days after treatment. The cells were washed with cold DPBS and scraped off from 100 mm Petri dishes. The nuclear extracts were prepared as previously described (Méndez and Stillman 2000). Briefly, the cells were extracted in cytosolic lysis buffer (10 mM HEPES, 10 mM KCl, 1.5 mM MgCl_2_, 0.34 M sucrose, 10% glycerol, and 1 mM DTT) supplemented with protease inhibitor cocktail (Sigma, P8340) and PMSF (Sigma, P7626) on ice for 10 min. Triton X-100 was then added to a final concentration of 0.1%. Cells were incubated on ice for another 5 min, and nuclei were collected by centrifugation at 1300 g for 4 min at 4 °C. The nuclei were washed once with cytosolic lysis buffer and extracted by nuclear lysis buffer (50 mM Tris pH 8.0, 150 mM NaCl, 1% NP40) supplemented with protease inhibitor cocktail and PMSF on ice for 10 min. The precipitates were removed by centrifugation at 17000 g for 10 min. The supernatant nuclear extracts were quantified by BCA assay (Beyotime, P0009) and used for gel filtration. For gel filtration, a superdex 200 increase 10/300 GL column (Cytiva, 28,990,944) pre-equilibrated in 50 mM Tris pH 8.0 and 150 mM NaCl was used. 500 μL of mESCs nuclear extracts containing 2 mg protein were loaded and 250-μL fractions were collected on an automated protein purifier (Union-Biotech, UEV 25 L). The input extract and fractions were then examined by Western blot.

### Analysis of 2C::tdTomato reporter mESCs

2C::tdTomato reporter mESCs were infected with lentiviruses carrying different shRNAs. The cells were then selected with Puromycin (2 μg/mL) for 2 days. Images were collected on MSHOT microscope with a 20x objective using MSHOT Image Analysis System v1.5.3 (MSHOT). To calculate the proportion of tdTomato-positive cells, we selected fields with 150–350 cells and counted the total cell number and the number of tdTomato-positive cells in each field. All experiments were performed three or more times, and representative results were shown.

### RNA isolation, qPCR, and RNA-seq

Total RNA was isolated from cells using the GeneJet RNA purification kit (Thermo Scientific, k0731), and 1 μg total RNA was reverse transcribed to generate cDNA using the PrimeScript RT reagent Kit (Takara, RR037A). The cDNA was then used as templates and qPCR analyses were performed using the SYBR Green qPCR Master Mix (Solomon Bio, QST-100) on the QuantStudio 3 Real-Time PCR system (Applied Biosystems). Primers used in qPCR experiments were listed in Supplementary Table 5. For RNA-seq, libraries were prepared from two biological replicates using the TruSeq RNA Sample Prep Kit and sequenced on the NextSeq (Illumina).

The RNA-seq data was subjected to quality check and adapter trimming by Trim Galore v0.6.4_dev and mapped to the mm9 reference genome using STAR v2.7.2d (Dobin et al. 2013). Reads summarization for genomic features was performed using featureCounts v2.0.0 (Liao et al. 2014). Reads normalization and differential expression were determined by DESeq2 v1.34.0 (for duplicate samples) and edgeR v3.36.0 (for single sample) (Love et al. 2014; Robinson et al. 2010). Batch effect correction was done using R package sva v3.20.0 (Leek et al. 2012). Pearson correlation coefficient among different samples was calculated using pre-determined fold changes (vs. control/wild type) by stats v4.1.3 in R. GO enrichment analysis was conducted using the enrichGO function in R package clusterProfiler v4.2.2 (Wu et al. 2021). Gene set enrichment analysis (GSEA) was performed with GSEA v4.2.3 (Subramanian et al. 2005). The 2C gene set was adopted from our previous study (Li et al. 2017).

### ChIP-seq

ChIP-seq was performed as described previously (Wang et al. 2014). Briefly, mESCs of 80–90% confluency were crosslinked with a final concentration of 1% formaldehyde for 10 min at room temperature, and then quenched by the addition of 125 mM glycine. The cells were then rinsed twice with ice-cold DPBS, and harvested by scraping using silicon scraper. The supernatant was discarded after spinning at 1350 g for 5 min at 4 °C, and the pellet was lysed with lysis buffer A (50 mM HEPES-KOH, pH 7.5, 140 mM NaCl, 1 mM EDTA, 0.5% NP-40, 0.25% Triton X-100, 10% glycerol and protease inhibitor cocktail), incubated at 4 °C for 10 min with rotating, and collected by spinning at 1350 g for 5 min at 4 °C. Cells were then resuspended in lysis buffer B (10 mM Tris-HCl, pH 8.0, 200 mM NaCl, 1 mM EDTA, 0.5 mM EGTA, and protease inhibitor cocktail), and incubated at room temperature for 10 min. The nuclei were then pelleted by spinning at 1350 g for 5 min at 4 °C. The pellet was resuspended with lysis buffer C (10 mM Tris-HCl, pH 8.0, 100 mM NaCl, 1 mM EDTA, 0.5 mM EGTA, 0.1% Na-Deoxycholate, 0.5% N-lauroylsarcosine, and protease inhibitor cocktail), incubated for 15 min on ice, and transferred into Covaris microTUBEs. The DNA was sonicated to 200 bp fragments using Covaris S220 (duty cycle: 10%; intensity: 5; cycles/burst: 200; duration: 195 s). After sonication, a final concentration of 1% Triton X-100 was added and gently mixed by pipetting. The chromatin solution was clarified by spinning at 12000 rpm at 4 °C for 10 min. 50 μL of supernatant from each sample was reserved as input and the rest chromatin solution was incubated with the indicated primary antibodies (anti-HA (for Pcgf6, CST, 3724S), anti-RNF2 (Active Motif, 39,663), and anti-H2AK119ub (CST, 8240S)) overnight at 4 °C. The magnetic beads (Thermo, 88,803) were added after blocking in the block buffer (0.5% (w/v) RNase/DNase-free BSA in 1 × PBS), and incubated with chromatin solution and antibodies for 4 h at 4 °C. The immunoprecipitant was then washed 5 times with wash buffer (50 mM HEPES-KOH, pH 7.6, 500 mM LiCl, 1 mM EDTA, 1% NP-40, 0.7% Na-deoxycholate). The DNA was then eluted with Elution Buffer (50 mM Tris-HCl, pH 8.0, 10 mM EDTA, 1% SDS). To reverse the crosslinks, samples were incubated at 65 °C overnight. The RNA was digested with a final concentration of 1 mg/mL RNases A at 37 °C for 1 h, and the proteins were degraded subsequently with a final concentration of 0.5 mg/mL Proteinase K at 55 °C for 2 h. The immunoprecipitated DNA was then extracted with AMPure beads according to the manufacturer’s instructions (Beckman, A63881). For ChIP-seq, 1 ng precipitated DNA or input was used to generate the DNA library using NEBNext Ultra II DNA Library Prep Kit for Illumina (NEB, E7645S). The libraries were sequenced by Next-Seq (Illumina).

The adapters and low-quality bases in the ChIP-seq raw data were removed using Trim Galore v0.6.4_dev, and the ChIP-seq reads were mapped to the mm9 genome using Bowtie2 v2.3.5.1 (Langmead and Salzberg 2012). Samtools v1.10 (Li et al. 2009) was used to filter and convert file formats, and MarkDuplicates v4.1.4.1 (Picard) was used to tag and remove the PCR duplicate reads (McKenna et al. 2010). The duplicates were removed using the “--REMOVE_SEQUENCING_DUPLICATES” option. ChIP-seq peaks were called using MACS2 v2.2.6 (Zhang et al. 2008). Visualization of genomic data was achieved by deepTools2 v3.3.1 and IGV v2.8.4 (Ramirez et al. 2016; Robinson et al. 2011). For the repetitive elements, the reads were aligned to the mm9 genome assembly using STAR v2.7.2d with the options ‘--alignIntronMax 1 --alignEndsType EndToEnd’ as previously reported (Madsen et al. 2020). The parameter ‘--outFilterMultimapNmax 1’ was applied to include only the uniquely mapped reads. Duplicate reads were then removed using MarkDuplicates from gatk package v.4.1.4.1. Replicate samples were merged using the samtools v1.10.

### Quantification and statistical analysis

Three or more independent experiments were performed for each analysis. Statistical analysis was done using GraphPad Prism v8.2.1. *p* values < 0.05 were considered to be statistically significant (ns, not significant; * *p* < 0.05; ** *p* < 0.01; *** *p* < 0.001; **** *p* < 0.0001). *n* represented biological replicates, and error bars represented the SD.

## Additional files

Additional file 1: **Fig. S1.** Correlation in transcriptional changes caused by downregulation of Rif1 or PRC1 components. A Scatter plot comparing the Fold Changes (FC) of gene expressions between Rif1 or RNF2 depleted mESCs. B Scatter plot comparing the FC of repetitive elements between Rif1 or RNF2 depleted mESCs. C-D Scatter plots comparing the FC of the expression of genes or repetitive elements between Rif1 or Pcgf6 depleted mESCs. The Pearson correlation coefficient (r) and the *p*-value are shown.

Additional file 2: **Supplementary Table 1.** The expression foldchanges of coding genes and repetitive elements.

Additional file 3: **Supplementary Table 2.** Summary of gene ontology (GO) analysis.

Additional file 4: **Supplementary Table 3.** Rif1, Pcgf6, RNF2, and H2AK119ub ChIP-seq peaks in mm9 genome.

Additional file 5: **Supplementary Table 4.** RNA-seq expression levels in Rif1 CKO and Pcgf6 KD mESCs.

Additional file 6: **Supplementary Table 5.** Sequences for primers, shRNAs, gRNAs and donors.

## Data Availability

The public datasets and that generated during the current study are listed in the tables below. All ChIP-seq (GSE203304) and RNA-seq (GSE203305) data in this paper are accessible at GEO: GSE203306 (https://www.ncbi.nlm.nih.gov/geo/query/acc.cgi?acc=GSE203306). Source codes and analysis pipelines have been uploaded to the GitHub pages (https://github.com/jlchen5/CELR-D-22-00015). All the plasmids and materials used in this study are available upon reasonable request.

## Abbreviations

mESCs: Mouse embryonic stem cells
2C: 2-cell
MERVL: Mouse endogenous retrovirus L
NHEJ: Non-homologous end joining
DSBs: DNA double strand breaks
PPI: Protein-protein interaction
GSEA: Gene set enrichment analysis
ICM: Inner cell mass
FC: Fold Changes
NES: Normalized enrichment scores
CKO: Conditional knockout
KI: Knock-in
GO: Gene ontology
LTR: Long terminal repeat
ChIP-seq: Chromatin immunoprecipitation followed by sequencing
RNA-seq: RNA sequencing
