# Supplementary figures and images for: Rif1 interacts with non-canonical polycomb repressive complex PRC1.6 to regulate mouse embryonic stem cells fate potential
Source: Cell Regen. 2022 Aug 2;11:25. doi: 10.1186/s13619-022-00124-9 (PMC9343540; doi:10.1186/s13619-022-00124-9)

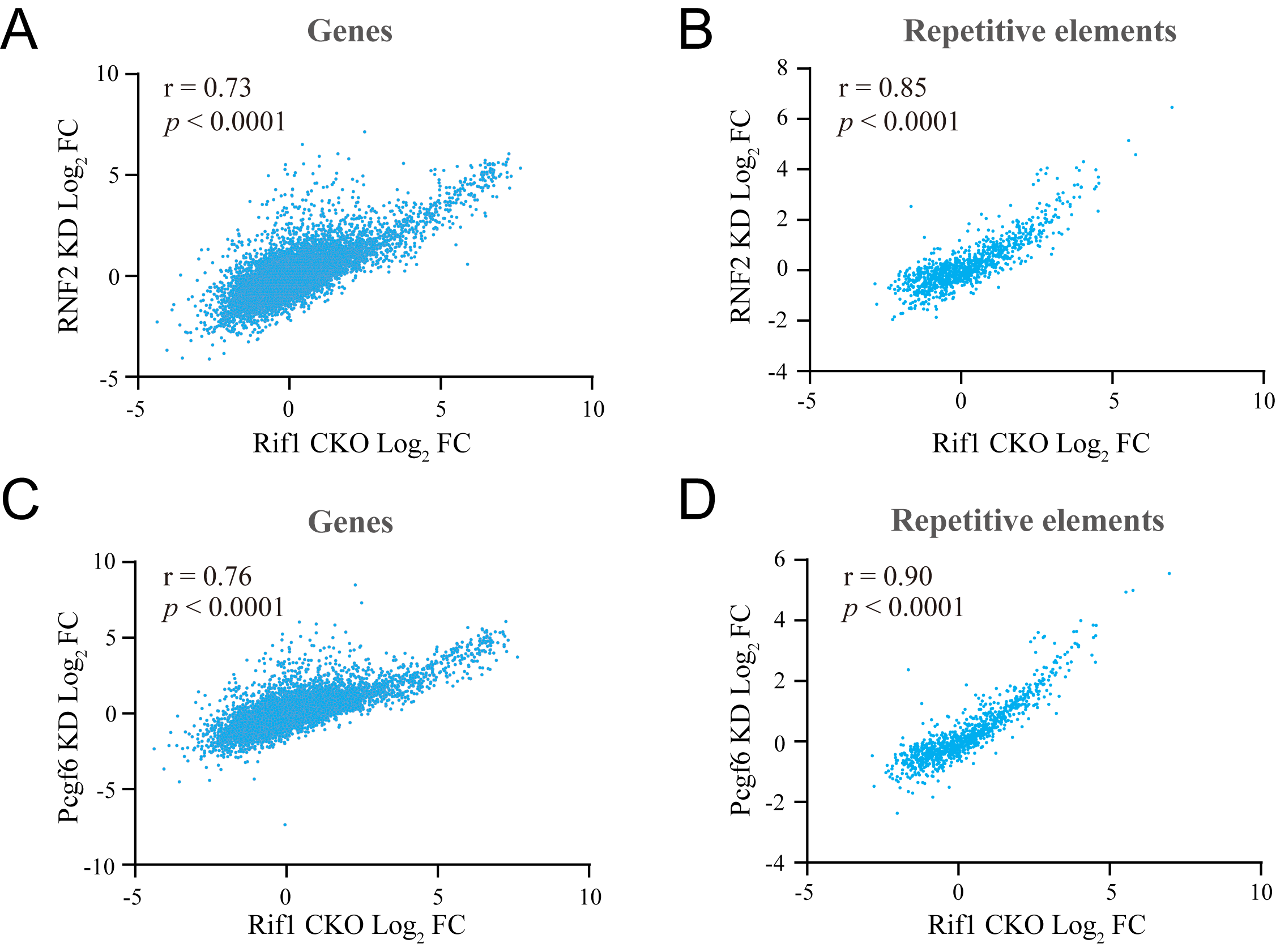

Supplement: Supplementary file 1 — Fig. S1. Correlation in transcriptional changes caused by downregulation of Rif1 or PRC1 components. A Scatter plot comparing the Fold Changes (FC) of gene expressions between Rif1 or RNF2 depleted mESCs. B Scatter plot comparing the FC of repetitive elements between Rif1 or RNF2 depleted mESCs. C-D Scatter plots comparing the FC of the expression of genes or repetitive elements between Rif1 or Pcgf6 depleted mESCs. The Pearson correlation coefficient (r) and the p-value are shown. [file 13619_2022_124_MOESM1_ESM.tif]
